# Supplementary material for: A screening of the MMV Pathogen Box® reveals new potential antifungal drugs against the etiologic agents of chromoblastomycosis
Source: PLoS One. 2020 May 13;15(5):e0229630. doi: 10.1371/journal.pone.0229630 (PMC7219733; doi:10.1371/journal.pone.0229630)
Supplement: S1 Table — Results are presented as the percent inhibition of growth (%IG) of the fungal strain. (PDF) [file pone.0229630.s001.pdf]

Plate	Code	ID	Disease	Compound	% Growth inhibition
A	MMV010764	A2	MALARIA	1	15.38461538
A	MMV1110498	B2	WOLBACHIA LF	2	13.46153846
A	MMV084603	C2	MALARIA	3	34.61538462
A	MMV687762	D2	KINETOPLASTID	4	34.61538462
A	MMV688514	E2	KINETOPLASTID	5	1.923076923
A	MMV026020	F2	MALARIA	6	30
A	MMV688470	G2	TOXOPLASMOSIS	7	37.69230769
A	MMV688350	H2	DENGUE	8	19.61538462
A	MMV688472	A3	TOXOPLASMOSIS	9	23.46153846
A	MMV000907	B3	MALARIA	10	26.92307692
A	MMV688548	C3	TOXOPLASMOSIS	11	25
A	MMV1028806	D3	MALARIA	12	39.23076923
A	MMV676350	E3	MALARIA	13	28.46153846
A	MMV688471	F3	TOXOPLASMOSIS	14	-22.30769231
A	MMV688704	G3	TOXOPLASMOSIS	15	23.07692308
A	MMV688360	H3	KINETOPLASTID	16	13.84615385
A	MMV688416	A4	DENGUE	17	29.61538462
A	MMV688889	B4	TUBERCULOSIS	18	24.23076923
A	MMV688888	C4	TUBERCULOSIS	19	8.076923077
A	MMV661713	D4	TUBERCULOSIS	20	30
A	MMV553002	E4	TUBERCULOSIS	21	17.30769231
A	MMV676388	F4	TUBERCULOSIS	22	27.69230769
A	MMV188296	G4	KINETOPLASTID	23	37.30769231
A	MMV099637	H4	KINETOPLASTID	24	31.92307692
A	MMV689758	A5	REFERENCE	25	28.84615385
A	MMV688776	B5	KINETOPLASTID	26	31.15384615
A	MMV690028	C5	KINETOPLASTID	27	21.15384615
A	MMV688793	D5	KINETOPLASTID	28	32.69230769
A	MMV688797	E5	KINETOPLASTID	29	12.30769231
A	MMV202553	F5	KINETOPLASTID	30	35.38461538
A	MMV688958	G5	KINETOPLASTID	31	19.61538462
A	MMV688798	H5	KINETOPLASTID	32	27.69230769
A	MMV688796	A6	KINETOPLASTID	33	7.692307692
A	MMV688934	B6	KINETOPLASTID	34	49.23076923
A	MMV688943	C6	KINETOPLASTID	35	98.46153846
A	MMV688942	D6	KINETOPLASTID	36	98.07692308
A	MMV688756	E6	TUBERCULOSIS	37	15.38461538
A	MMV688936	F6	TUBERCULOSIS	38	24.23076923
A	MMV063404	G6	TUBERCULOSIS	39	39.23076923
A	MMV676539	H6	TUBERCULOSIS	40	40.76923077
A	MMV676526	A7	TUBERCULOSIS	41	20
A	MMV676389	B7	TUBERCULOSIS	42	23.46153846
A	MMV053220	C7	TUBERCULOSIS	43	5.769230769
A	MMV688554	D7	TUBERCULOSIS	44	25.76923077
A	MMV090930	E7	TUBERCULOSIS	45	-1.153846154
A	MMV676476	F7	TUBERCULOSIS	46	27.30769231

A	MMV676558	G7	TUBERCULOSIS	47	26.92307692
A	MMV202458	H7	TUBERCULOSIS	48	3.846153846
A	MMV688553	A8	TUBERCULOSIS	49	26.92307692
A	MMV676603	B8	TUBERCULOSIS	50	19.23076923
A	MMV676584	C8	TUBERCULOSIS	51	25
A	MMV676555	D8	TUBERCULOSIS	52	29.23076923
A	MMV676431	E8	TUBERCULOSIS	53	10.76923077
A	MMV676377	F8	TUBERCULOSIS	54	33.84615385
A	MMV688555	G8	TUBERCULOSIS	55	11.53846154
A	MMV676474	H8	TUBERCULOSIS	56	17.30769231
A	MMV676501	A9	TUBERCULOSIS	57	41.15384615
A	MMV676401	B9	TUBERCULOSIS	58	31.92307692
A	MMV676439	C9	TUBERCULOSIS	59	23.46153846
A	MMV676383	D9	TUBERCULOSIS	60	21.15384615
A	MMV676571	E9	TUBERCULOSIS	61	14.61538462
A	MMV676406	F9	TUBERCULOSIS	62	26.92307692
A	MMV676597	G9	TUBERCULOSIS	63	25
A	MMV461553	H9	TUBERCULOSIS	64	13.84615385
A	MMV676449	A10	TUBERCULOSIS	65	8.076923077
A	MMV102872	B10	TUBERCULOSIS	66	36.53846154
A	MMV676395	C10	TUBERCULOSIS	67	25
A	MMV676444	D10	TUBERCULOSIS	68	19.61538462
A	MMV676445	E10	TUBERCULOSIS	69	30.38461538
A	MMV676461	F10	TUBERCULOSIS	70	36.53846154
A	MMV676588	G10	TUBERCULOSIS	71	16.92307692
A	MMV676520	H10	TUBERCULOSIS	72	10.76923077
A	MMV676412	A11	TUBERCULOSIS	73	13.84615385
A	MMV676477	B11	TUBERCULOSIS	74	25.76923077
A	MMV676379	C11	TUBERCULOSIS	75	-40
A	MMV676409	D11	TUBERCULOSIS	76	30.38461538
A	MMV676589	E11	TUBERCULOSIS	77	7.692307692
A	MMV676509	F11	TUBERCULOSIS	78	7.692307692
A	MMV676554	G11	TUBERCULOSIS	79	26.53846154
A	MMV676512	H11	TUBERCULOSIS	80	-12.69230769
B	MMV676480	A2	ONCHOCERCIASIS	81	29.08496732
B	MMV012074	B2	TUBERCULOSIS	82	37.90849673
B	MMV690027	C2	KINETOPLASTID	83	50.98039216
B	MMV020537	D2	MALARIA	84	48.36601307
B	MMV676386	E2	TUBERCULOSIS	85	7.843137255
B	MMV019189	F2	MALARIA	86	40.8496732
B	MMV069458	G2	TUBERCULOSIS	87	37.25490196
B	MMV676602	H2	KINETOPLASTID	88	37.25490196
B	MMV652003	A3	KINETOPLASTID	89	35.94771242
B	MMV676604	B3	KINETOPLASTID	90	33.9869281
B	MMV676600	C3	KINETOPLASTID	91	25.49019608
B	MMV637953	D3	REFERENCE	92	51.96078431
B	MMV688773	E3	REFERENCE	93	28.75816993

B	MMV688774	F3	REFERENCE	94	99.34640523
B	MMV688991	G3	REFERENCE	95	40.8496732
B	MMV000016	H3	REFERENCE	96	34.96732026
B	MMV000062	A4	REFERENCE	97	49.67320261
B	MMV002529	B4	REFERENCE	98	36.92810458
B	MMV676382	C4	SCHISTOSOMIASIS	99	41.83006536
B	MMV676536	D4	SCHISTOSOMIASIS	100	21.56862745
B	MMV000011	E4	REFERENCE	101	25.81699346
B	MMV003270	F4	ANCYLOSTOMIASIS	102	21.56862745
B	MMV687801	G4	REFERENCE	103	29.41176471
B	MMV687803	H4	REFERENCE	104	29.73856209
B	MMV006372	A5	MALARIA	105	41.83006536
B	MMV687776	B5	LYMPHATIC FILARIASIS	106	0.980392157
B	MMV001625	C5	REFERENCE	107	28.43137255
B	MMV000063	D5	REFERENCE	108	33.33333333
B	MMV687775	E5	LYMPHATIC FILARIASIS	109	42.81045752
B	MMV637229	F5	TRICHURIASIS	110	39.21568627
B	MMV689480	G5	REFERENCE	111	29.08496732
B	MMV668727	H5	ONCHOCERCIASIS	112	30.06535948
B	MMV688854	A6	CRYPTOSPORIDIUM INFECTION	113	30.39215686
B	MMV687800	B6	REFERENCE	114	33.33333333
B	MMV001493	C6	ONCHOCERCIASIS	115	34.31372549
B	MMV689255	D6	CRYPTOSPORIDIUM INFECTION	116	23.85620915
B	MMV002817	E6	ONCHOCERCIASIS	117	99.67320261
B	MMV688853	F6	CRYPTOSPORIDIUM INFECTION	118	37.58169935
B	MMV003152	G6	REFERENCE	119	39.54248366
B	MMV019742	H6	MALARIA	120	27.77777778
B	MMV011903	A7	MALARIA	121	21.89542484
B	MMV020982	B7	MALARIA	122	20.91503268
B	MMV020136	C7	MALARIA	123	25.16339869
B	MMV019838	D7	MALARIA	124	26.14379085
B	MMV676442	E7	MALARIA	125	17.97385621
B	MMV020321	F7	MALARIA	126	28.75816993
B	MMV006239	G7	MALARIA	127	35.62091503
B	MMV009054	H7	MALARIA	128	31.04575163
B	MMV020591	A8	MALARIA	129	26.79738562
B	MMV020120	B8	MALARIA	130	31.04575163
B	MMV020710	C8	MALARIA	131	32.35294118
B	MMV020520	D8	MALARIA	132	41.83006536
B	MMV020152	E8	MALARIA	133	35.94771242
B	MMV019087	F8	MALARIA	134	23.85620915
B	MMV000858	G8	MALARIA	135	37.25490196
B	MMV006901	H8	MALARIA	136	37.25490196
B	MMV020623	A9	MALARIA	137	16.66666667
B	MMV676605	B9	MALARIA	138	8.496732026
B	MMV020517	C9	MALARIA	139	33.00653595
B	MMV019234	D9	MALARIA	140	6.535947712

B	MMV024397	E9	MALARIA	141	26.79738562
B	MMV676528	F9	MALARIA	142	33.33333333
B	MMV006741	G9	MALARIA	143	25.16339869
B	MMV020391	H9	MALARIA	144	25.81699346
B	MMV020512	A10	MALARIA	145	13.07189542
B	MMV007638	B10	MALARIA	146	34.31372549
B	MMV019721	C10	MALARIA	147	36.60130719
B	MMV016136	D10	MALARIA	148	20.26143791
B	MMV019807	E10	MALARIA	149	25.16339869
B	MMV020320	F10	MALARIA	150	20.26143791
B	MMV688768	G10	SCHISTOSOMIASIS	151	23.85620915
B	MMV676380	H10	MALARIA	152	41.83006536
B	MMV688761	A11	SCHISTOSOMIASIS	153	19.93464052
B	MMV021057	B11	MALARIA	154	94.77124183
B	MMV688763	C11	SCHISTOSOMIASIS	155	27.77777778
B	MMV688762	D11	SCHISTOSOMIASIS	156	15.35947712
B	MMV560185	E11	MALARIA	157	45.4248366
B	MMV085210	F11	MALARIA	158	25.49019608
B	MMV000023	G11	REFERENCE	159	27.77777778
B	MMV688994	H11	REFERENCE	160	26.79738562
C	MMV675997	A2	KINETOPLASTID	161	36.42384106
C	MMV020388	B2	MALARIA	162	38.0794702
C	MMV688508	C2	TUBERCULOSIS	163	4.966887417
C	MMV688410	D2	KINETOPLASTID	164	31.78807947
C	MMV675994	E2	CRYPTOSPORIDIUM INFECTION	165	19.53642384
C	MMV676053	F2	CRYPTOSPORIDIUM INFECTION	166	27.15231788
C	MMV676191	G2	CRYPTOSPORIDIUM INFECTION	167	38.74172185
C	MMV675969	H2	ONCHOCERCIASIS	168	31.12582781
C	MMV676204	A3	ONCHOCERCIASIS	169	44.37086093
C	MMV688547	B3	KINETOPLASTID	170	19.53642384
C	MMV688283	C3	KINETOPLASTID	171	30.79470199
C	MMV676048	D3	KINETOPLASTID	172	28.47682119
C	MMV676057	E3	KINETOPLASTID	173	27.48344371
C	MMV688179	F3	KINETOPLASTID	174	72.84768212
C	MMV675993	G3	CRYPTOSPORIDIUM INFECTION	175	30.79470199
C	MMV688313	H3	SCHISTOSOMIASIS	176	41.05960265
C	MMV687239	A4	TUBERCULOSIS	177	40.06622517
C	MMV688466	B4	TUBERCULOSIS	178	28.80794702
C	MMV687243	C4	TUBERCULOSIS	179	20.52980132
C	MMV687703	D4	TUBERCULOSIS	180	5.629139073
C	MMV687699	E4	TUBERCULOSIS	181	26.49006623
C	MMV023969	F4	TUBERCULOSIS	182	22.51655629
C	MMV021660	G4	TUBERCULOSIS	183	26.49006623
C	MMV687172	H4	TUBERCULOSIS	184	44.70198675
C	MMV688122	A5	TUBERCULOSIS	185	40.06622517
C	MMV687749	B5	TUBERCULOSIS	186	32.45033113
C	MMV687730	C5	TUBERCULOSIS	187	15.23178808

C	MMV687248	D5	TUBERCULOSIS	188	35.43046358
C	MMV687146	E5	TUBERCULOSIS	189	21.85430464
C	MMV687138	F5	TUBERCULOSIS	190	32.45033113
C	MMV688417	G5	TOXOPLASMOSIS	191	41.7218543
C	MMV688844	H5	TUBERCULOSIS	192	32.1192053
C	MMV688852	A6	TOXOPLASMOSIS	193	32.78145695
C	MMV688846	B6	TUBERCULOSIS	194	32.78145695
C	MMV687251	C6	TUBERCULOSIS	195	35.43046358
C	MMV688125	D6	TUBERCULOSIS	196	34.10596026
C	MMV687696	E6	TUBERCULOSIS	197	12.25165563
C	MMV688262	F6	TUBERCULOSIS	198	30.46357616
C	MMV687273	G6	TUBERCULOSIS	199	37.41721854
C	MMV1198433	H6	SCHISTOSOMIASIS	200	26.49006623
C	MMV687145	A7	TUBERCULOSIS	201	38.0794702
C	MMV054312	B7	TUBERCULOSIS	202	33.11258278
C	MMV687254	C7	TUBERCULOSIS	203	34.43708609
C	MMV687188	D7	TUBERCULOSIS	204	31.12582781
C	MMV687170	E7	TUBERCULOSIS	205	27.48344371
C	MMV687189	F7	TUBERCULOSIS	206	20.1986755
C	MMV687180	G7	TUBERCULOSIS	207	29.8013245
C	MMV024311	H7	TUBERCULOSIS	208	34.76821192
C	MMV688327	A8	TUBERCULOSIS	209	20.1986755
C	MMV689060	B8	KINETOPLASTID	210	24.17218543
C	MMV688509	C8	TOXOPLASMOSIS	211	37.41721854
C	MMV690103	D8	KINETOPLASTID	212	20.86092715
C	MMV690102	E8	KINETOPLASTID	213	20.86092715
C	MMV687807	F8	TUBERCULOSIS	214	13.24503311
C	MMV1088520	G8	MALARIA	215	29.13907285
C	MMV1019989	H8	MALARIA	216	21.85430464
C	MMV008439	A9	MALARIA	217	33.77483444
C	MMV689061	B9	KINETOPLASTID	218	27.48344371
C	MMV688361	C9	KINETOPLASTID	219	31.45695364
C	MMV688124	D9	TUBERCULOSIS	220	23.50993377
C	MMV689709	E9	KINETOPLASTID	221	26.82119205
C	MMV676478	F9	TUBERCULOSIS	222	36.09271523
C	MMV688891	G9	TUBERCULOSIS	223	35.09933775
C	MMV1037162	H9	MALARIA	224	20.1986755
C	MMV595321	A10	KINETOPLASTID	225	34.10596026
C	MMV689028	B10	KINETOPLASTID	226	31.78807947
C	MMV689029	C10	KINETOPLASTID	227	32.1192053
C	MMV688845	D10	TUBERCULOSIS	228	23.8410596
C	MMV021375	E10	MALARIA	229	15.89403974
C	MMV062221	F10	MALARIA	230	43.04635762
C	MMV023370	G10	MALARIA	231	18.54304636
C	MMV689437	H10	KINETOPLASTID	232	38.74172185
C	MMV687747	A11	TUBERCULOSIS	233	29.8013245
C	MMV688371	B11	KINETOPLASTID	234	34.76821192

C	MMV022236	C11	MALARIA	235	21.19205298
C	MMV1030799	D11	MALARIA	236	33.11258278
C	MMV1029203	E11	MALARIA	237	37.08609272
C	MMV688921	F11	DENGUE	238	47.01986755
C	MMV688703	G11	TOXOPLASMOSIS	239	42.05298013
C	MMV688955	H11	TOXOPLASMOSIS	240	22.18543046
D	MMV026468	A2	MALARIA	241	24.50331126
D	MMV011511	B2	MALARIA	242	31.78807947
D	MMV020291	C2	MALARIA	243	36.09271523
D	MMV676269	D2	MALARIA	244	28.80794702
D	MMV023233	E2	MALARIA	245	20.52980132
D	MMV009135	F2	MALARIA	246	25.49668874
D	MMV007803	G2	MALARIA	247	42.38410596
D	MMV007133	H2	MALARIA	248	25.82781457
D	MMV020670	A3	MALARIA	249	33.44370861
D	MMV007625	B3	MALARIA	250	34.43708609
D	MMV006833	C3	MALARIA	251	8.278145695
D	MMV020081	D3	MALARIA	252	36.75496689
D	MMV085230	E3	MALARIA	253	34.10596026
D	MMV011765	F3	MALARIA	254	40.39735099
D	MMV001059	G3	MALARIA	255	20.52980132
D	MMV022478	H3	MALARIA	256	53.31125828
D	MMV023953	A4	MALARIA	257	22.51655629
D	MMV007471	B4	MALARIA	258	-3.973509934
D	MMV026490	C4	MALARIA	259	27.15231788
D	MMV026550	D4	MALARIA	260	33.77483444
D	MMV085071	E4	MALARIA	261	17.21854305
D	MMV024937	F4	MALARIA	262	20.1986755
D	MMV011691	G4	MALARIA	263	41.05960265
D	MMV024101	H4	MALARIA	264	47.01986755
D	MMV010576	A5	MALARIA	265	34.10596026
D	MMV024829	B5	MALARIA	266	29.8013245
D	MMV687246	C5	MALARIA	267	14.90066225
D	MMV675995	D5	ONCHOCERCIASIS	268	32.1192053
D	MMV659004	E5	KINETOPLASTID	269	53.64238411
D	MMV085499	F5	MALARIA	270	36.42384106
D	MMV676877	G5	MALARIA	271	42.38410596
D	MMV676881	H5	MALARIA	272	39.73509934
D	MMV032967	A6	MALARIA	273	37.74834437
D	MMV045105	B6	KINETOPLASTID	274	33.77483444
D	MMV676162	C6	KINETOPLASTID	275	19.20529801
D	MMV688274	D6	KINETOPLASTID	276	32.1192053
D	MMV676260	E6	MALARIA	277	18.54304636
D	MMV023985	F6	MALARIA	278	36.09271523
D	MMV663250	G6	MALARIA	279	53.31125828
D	MMV024443	H6	MALARIA	280	47.35099338
D	MMV031011	A7	MALARIA	281	25.82781457

D	MMV022029	B7	MALARIA	282	30.79470199
D	MMV024114	C7	MALARIA	283	38.0794702
D	MMV023860	D7	MALARIA	284	31.78807947
D	MMV688364	E7	TOXOPLASMOSIS	285	25.49668874
D	MMV024195	F7	MALARIA	286	39.07284768
D	MMV407539	G7	WOLBACHIA LF	287	24.17218543
D	MMV688469	H7	TOXOPLASMOSIS	288	39.07284768
D	MMV688178	A8	SCHISTOSOMIASIS	289	25.16556291
D	MMV676064	B8	ONCHOCERCIASIS	290	36.42384106
D	MMV688467	C8	KINETOPLASTID	291	40.39735099
D	MMV688407	D8	KINETOPLASTID	292	2.317880795
D	MMV032995	E8	MALARIA	293	35.7615894
D	MMV676063	F8	ONCHOCERCIASIS	294	25.16556291
D	MMV688372	G8	KINETOPLASTID	295	37.74834437
D	MMV023388	H8	MALARIA	296	31.12582781
D	MMV688362	A9	KINETOPLASTID	297	26.82119205
D	MMV688180	B9	KINETOPLASTID	298	35.43046358
D	MMV675998	C9	KINETOPLASTID	299	6.622516556
D	MMV023949	D9	MALARIA	300	24.50331126
D	MMV688279	E9	KINETOPLASTID	301	36.09271523
D	MMV676186	F9	KINETOPLASTID	302	49.00662252
D	MMV658993	G9	KINETOPLASTID	303	29.47019868
D	MMV675968	H9	CRYPTOSPORIDIUM INFECTION	304	36.42384106
D	MMV687706	A10	KINETOPLASTID	305	26.82119205
D	MMV024035	B10	MALARIA	306	38.74172185
D	MMV659010	C10	KINETOPLASTID	307	34.43708609
D	MMV676050	D10	CRYPTOSPORIDIUM INFECTION	308	23.17880795
D	MMV688271	E10	KINETOPLASTID	309	36.42384106
D	MMV688474	F10	KINETOPLASTID	310	41.7218543
D	MMV676182	G10	CRYPTOSPORIDIUM INFECTION	311	34.43708609
D	MMV675996	H10	ONCHOCERCIASIS	312	22.51655629
D	MMV026356	A11	KINETOPLASTID	313	39.07284768
D	MMV688941	B11	TUBERCULOSIS	314	33.44370861
D	MMV676008	C11	KINETOPLASTID	315	18.87417219
D	MMV024406	D11	MALARIA	316	27.48344371
D	MMV019790	E11	MALARIA	317	29.8013245
D	MMV687812	F11	TUBERCULOSIS	318	23.50993377
D	MMV676411	G11	TUBERCULOSIS	319	11.25827815
D	MMV688980	H11	MALARIA	320	11.25827815
E	MMV011229	A2	MALARIA	321	5.531914894
E	MMV687794	B2	MALARIA	322	4.680851064
E	MMV676398	C2	WOLBACHIA LF	323	23.40425532
E	MMV688766	D2	SCHISTOSOMIASIS	324	21.70212766
E	MMV020289	E2	MALARIA	325	8.936170213
E	MMV019551	F2	MALARIA	326	8.936170213
E	MMV146306	G2	TUBERCULOSIS	327	37.0212766
E	MMV688330	H2	TOXOPLASMOSIS	328	15.74468085

E	MMV676468	A3	TUBERCULOSIS	329	28.08510638
E	MMV676470	B3	TUBERCULOSIS	330	18.29787234
E	MMV676472	C3	TUBERCULOSIS	331	11.91489362
E	MMV200748	D3	TUBERCULOSIS	332	31.4893617
E	MMV002816	E3	REFERENCE	333	14.89361702
E	MMV688552	F3	SCHISTOSOMIASIS	334	0.85106383
E	MMV688557	G3	TUBERCULOSIS	335	43.40425532
E	MMV687796	H3	REFERENCE	336	11.4893617
E	MMV688771	A4	SCHISTOSOMIASIS	337	13.61702128
E	MMV688938	B4	TUBERCULOSIS	338	20.42553191
E	MMV671636	C4	ONCHOCERCIASIS	339	25.10638298
E	MMV667494	D4	MALARIA	340	27.23404255
E	MMV634140	E4	MALARIA	341	7.659574468
E	MMV016838	F4	MALARIA	342	16.59574468
E	MMV021013	G4	TUBERCULOSIS	343	61.2766
E	MMV688939	H4	TUBERCULOSIS	344	-4.680851064
E	MMV687798	A5	REFERENCE	345	16.17021277
E	MMV689000	B5	REFERENCE	346	18.72340426
E	MMV676599	C5	CRYPTOSPORIDIUM INFECTION	347	-7.659574468
E	MMV028694	D5	MALARIA	348	16.59574468
E	MMV030734	E5	MALARIA	349	17.44680851
E	MMV676270	F5	MALARIA	350	2.978723404
E	MMV392832	G5	MALARIA	351	9.787234043
E	MMV688978	H5	REFERENCE	352	81.70212766
E	MMV688775	A6	REFERENCE	353	19.57446809
E	MMV004168	B6	KINETOPLASTID	354	11.4893617
E	MMV689244	C6	KINETOPLASTID	355	8.510638298
E	MMV001499	D6	REFERENCE	356	8.510638298
E	MMV689243	E6	KINETOPLASTID	357	8.510638298
E	MMV688755	F6	TUBERCULOSIS	358	5.106382979
E	MMV688754	G6	KINETOPLASTID	359	82.12765957
E	MMV688990	H6	REFERENCE	360	21.70212766
E	MMV676159	A7	KINETOPLASTID	361	6.808510638
E	MMV676161	B7	KINETOPLASTID	362	-10.21276596
E	MMV688411	C7	TOXOPLASMOSIS	363	5.957446809
E	MMV688345	D7	TOXOPLASMOSIS	364	11.4893617
E	MMV676358	E7	MALARIA	365	22.55319149
E	MMV228911	F7	TUBERCULOSIS	366	7.234042553
E	MMV001561	G7	KINETOPLASTID	367	11.06382979
E	MMV688273	H7	KINETOPLASTID	368	5.957446809
E	MMV393144	A8	MALARIA	369	15.31914894
E	MMV023183	B8	MALARIA	370	31.06382979
E	MMV687765	C8	TUBERCULOSIS	371	26.80851064
E	MMV010545	D8	MALARIA	372	17.87234043
E	MMV687729	E8	TUBERCULOSIS	373	3.404255319
E	MMV272144	F8	TUBERCULOSIS	374	40.85106383
E	MMV658988	G8	KINETOPLASTID	375	20

E	MMV393995	H8	TUBERCULOSIS	376	1.276595745
E	MMV007920	A9	MALARIA	377	17.0212766
E	MMV047015	B9	TUBERCULOSIS	378	8.085106383
E	MMV020165	C9	MALARIA	379	20.42553191
E	MMV023227	D9	MALARIA	380	17.44680851
E	MMV407834	E9	MALARIA	381	25.53191489
E	MMV026313	F9	MALARIA	382	9.787234043
E	MMV084864	G9	MALARIA	383	2.978723404
E	MMV1236379	H9	KINETOPLASTID	384	24.68085106
E	MMV688270	A10	SCHISTOSOMIASIS	385	13.61702128
E	MMV688795	B10	KINETOPLASTID	386	15.31914894
E	MMV676524	C10	TUBERCULOSIS	387	6.382978723
E	MMV687700	D10	TUBERCULOSIS	388	10.63829787
E	MMV687813	E10	TUBERCULOSIS	389	7.234042553
E	MMV161996	F10	TUBERCULOSIS	390	14.46808511
E	MMV676492	G10	LYMPHATIC FILARIASIS	391	22.55319149
E	MMV688550	H10	KINETOPLASTID	392	10.63829787
E	MMV019993	A11	MALARIA	393	16.59574468
E	MMV688352	B11	DENGUE	394	25.53191489
E	MMV611037	C11	TUBERCULOSIS	395	29.78723404
E	MMV676384	D11	TUBERCULOSIS	396	19.14893617
E	MMV153413	E11	TUBERCULOSIS	397	14.04255319
E	MMV688543	F11	DENGUE	398	11.06382979
E	MMV688415	G11	KINETOPLASTID	399	13.61702128
E	MMV495543	H11	TUBERCULOSIS	400	-3.829787234
