# Supplementary material for: A screening of the MMV Pathogen Box® reveals new potential antifungal drugs against the etiologic agents of chromoblastomycosis
Source: PLoS One. 2020 May 13;15(5):e0229630. doi: 10.1371/journal.pone.0229630 (PMC7219733; doi:10.1371/journal.pone.0229630)
Supplement: Attachment — Submitted filename: Response to Reviewers.doc [file pone.0229630.s003.doc]

**Laboratório de Micologia**

#### Av. Brasil, 4365, Manguinhos

**CEP 21045-900 - Rio de Janeiro –RJ -BRASIL**

**Fone: 55 (21) 3865-9642, Fax: 55 (21) 2590-9988**

e mail:rowena.alves@ini.fiocruz.br

April 7^th^, 2020

Dr. Kirsten Nielsen

Academic Editor, PLoS One

Dear Dr. Nielsen,

We sincerely appreciate the thoughtful review that you and the reviewers have provided for our manuscript **(**PONE-D-20-03641**)**. Addressing the constructive comments has improved our manuscript. In this new version of the manuscript we also placed figure legends and tables after the paragraphs they are first mentioned in the text, as requested by the PLoS One author guidelines. Our point-by-point responses to the comments are:

**Major points:**

Reviewer #1: L116: 1% DMSO seems like a high percentage of DMSO, and it could be inhibitory alone to the cells -- was a DMSO control included? If so, this should be included in the text, if not the experiments should be repeated to verify 1% DMSO is not inhibitory or justification for why it was not should be explained.

**Answer: We included a DMSO control on the test board, since we follow the EUCAST protocol that recommends a concentration of 1% DMSO during preparation of the microplates for susceptibility testing. After addition of the fungal inoculum, this concentration drops to 0.5%, which is the final DMSO concentration in the antifungal susceptibility testing. This information was clarified in the new version of the text (lines 122-124 and lines 127-131).**

Reviewer #1: Table 1: Please provide some clarification on why the MFC value is lower than the MIC value for auranofin against *P. verrucosa* and for bitertanol against *P. verrucosa*. And why they are still considered fungistatic? Denoting which drugs were fungicidal would also be helpful in this table, so they are readily identifiable.

**Answer: Thank you for this observation. The MFC lower than the MIC values are really intriguing, therefore we prepared two additional replicates using these compounds against the *P. verrucosa* isolate. We found MIC values lower or equal to the MFC values. We believe that this one-fold dilution variation on our MFC values were due to intrinsic aspects of the methodology, which has several pipetting and diluting steps that can induce manual errors in the measurements. In fact, several publications only consider two-fold variations on MIC/MFC values as significant. Again, thank you for this observation.**

Reviewer #1: L144 -145: “Compounds that presented a MFC value lower than two times the MIC were considered fungicidal.” I am confused by this and need more clarification, especially how this relates to the fungicide determination presented in Table 1. In the results (lines 195-196) the authors state that auranofin is fungicidal against *E. dermatidis* and iodoquinol is fungicidal against *P. verrucosa*, but the MFC and MIC values are the same. Perhaps explaining fungicidal determination in terms of MFC to MIC ratio would make it easier for the reader to understand and would be less open to interpretation.

**Answer: Thank you for this suggestion. We have changed the definition (lines 152-154) accordingly and also the MFC/MIC ratio was included in table 1 to facilitate the readers’ understanding. Results of fungicidal drugs were also updated in this new version of the manuscript (lines 224-228). Moreover, as mentioned before, we repeated the test twice to confirm the results after receiving the reviewer’s comments.**

**--------------------**

Reviewer #2: Is Auranofin fungistatic against *P. verrucosa*? Lines 195-196 say that “Auranofin (MMV688978) was fungicidal against the *E. dermatitidis* strain and 196 fungistatic against the other species”. The definition of a fungicidal drug on line 144 indicate that “Compounds that presented a MFC value lower than two times the MIC were considered fungicidal”. For *P. verrucosa* MIC=1.25 AND MFC=0.625. Unless I am mistaken I think this MFC value indicate that Auranofin is fungicidal against *P. verrucosa* too.

**Answer: We followed the helpful suggestion of reviewer #1 to address this problem. In the new version of our manuscript we corrected the results related to fungicidal effects of the evaluated drugs against *P. verrucosa* (lines 224-228).**

Reviewer #2: I will suggest to add what “CC50/CC20” means below Table 2.

**Answer: Thank you for this suggestion. These definitions were added in table 2 footnotes as requested.**

**--------------------**

**Minor points:**

Reviewer #1: Why was *F. pedrosoi* chosen as the strain to test initially? More background on CBM would be interesting to include in the introduction.

**Answer: We agree with the reviewer. A description of the species epidemiology on the major endemic areas was included in the first paragraph of introduction (Lines 59-65). In addition, the explanation for using this species in all assays, including the screening, was included in the “fungal strains and culture conditions” section of Materials and methods (Lines 95-96).**

Reviewer #1: L44-45: Include in the abstract why MMV021013 was thought to be promising. It is included for the other two, but not for the MMV021013.

**Answer #1: We added a sentence about this subject in lines 44-45.**

Reviewer #1: L65: delete “for”, so it reads “time to seek medical help”

**Answer #1: This was corrected as requested.**

Reviewer #1: L81: italicize in vitro

**Answer #1: This was corrected as requested.**

Reviewer #1: L148: Define CC50 in text.

**Answer #1: This definition was included in the new version of the manuscript (lines 157-158)**

Reviewer #1: L266 & 267: Keep spelling of amebicide consistent.

**Answer: This was corrected as requested.**

Reviewer #1: Figure 1: I liked that you the compounds were grouped by original application, but it would be nice if the 8 compounds that were studied further were labelled. The markers could also be made smaller just to make it easier to see all the markers.

**Answer: A revised Fig1 file was included in the new version of our manuscript. In this new version of Fig 1, we used colors to differentiate the compounds that were further studied. Also, we used smaller markers and increased the sizes of both x and y axis, to facilitate the visualization of the points. However, there are some classes of compounds (e.g. anti-malaria) with a lot of data points, so it is still difficult to differentiate most of them. But, in our opinion, in this new version of the figure, the compounds with anti-CBM activity are clearly differentiated. We sincerely wish that this new Fig1 file will please the reviewers.**

Reviewer #1: Figure 2: I am not sure this figure is necessary – perhaps it could be included as a supplementary figure.

**Answer: We preferred to maintain this figure within the manuscript, as usually practiced in similar studies. Moreover, reviewer #2 made a minor comment on our text about the chemical structure of the compounds, so we suppose that the other reviewer also thinks that this figure fits within the main text of our manuscript.**

Reviewer #1: Table 2, L478: Unsure what “* Cytotoxicity dada only CC20 Hep G2 cells” means. – Is “dada” supposed to be data?

**Answer: This legend was corrected (Line 291). Thanks for this observation.**

**--------------------**

Reviewer #2: Line 81, “in vitro” should be in italic
**Answer: This was corrected as requested.**

Reviewer #2: Line 182, add a period before “The chemical structure…”.

**Answer: This was corrected as requested.**

Reviewer #2: Line 478, correct “cytotoxicity dada”

**Answer: This was corrected as requested.**

*After the last review the changes suggested by reviewers # 1 and # 2 have been corrected. Thanks for this observation.

We are hopeful that the revised manuscript will be suitable for publication in PLoS One.

Sincerely,

Rowena Alves Coelho.
